# Notoginsenoside R1 Improved Hypoxic Pulmonary Hypertension by Inhibiting Glycolysis-Mediated Pulmonary Arterial Vascular Remodeling

**DOI:** 10.1155/carj/2884885

**Published:** 2025-10-13

**Authors:** Xiaowei Gong, Yanling Sheng, Gaijun Zhang, Shiwei Kang, Xin Liu, Yuming Wang, Tao Zhang, Hanzhou Li, Huan Pei, Yadong Yuan, Feitian Min, Huantian Cui

**Affiliations:** ^1^Department of Respiratory Medicine, The Second Hospital of Hebei Medical University, Shijiazhuang 050000, China; ^2^Department of Respiratory and Critical Care Medicine, Huabei Petroleum Administration Bureau General Hospital, Cangzhou 062550, China; ^3^First School of Clinical Medicine, Yunnan University of Chinese Medicine, Kunming 650500, China; ^4^School of Integrative Chinese and Western Medicine, Tianjin University of Traditional Chinese Medicine, Tianjin 301617, China

**Keywords:** glycolysis, hypoxic pulmonary hypertension, Notoginsenoside R1, transcriptomics, untargeted metabolomics

## Abstract

Hypoxic pulmonary hypertension (HPH) lacks effective treatments. The research is designed to examine the effectiveness of Notoginsenoside R1 (NGR1) in addressing HPH and to explore its molecular mechanisms. Under hypoxic conditions, we created a rat model of HPH and treated the animals with NGR1. We assessed the therapeutic effects of NGR1 on HPH through hemodynamic measurements and pulmonary artery vascular remodeling. We employed transcriptomic analysis to evaluate gene expression changes in HPH rats. We conducted untargeted metabolomics to examine how NGR1 influences the metabolic profile of HPH rats. NGR1 treatment significantly improved hemodynamic parameters and ameliorated pulmonary artery vascular remodeling in HPH rats. Transcriptomic analysis identified *Pck1* as the most significantly altered gene. NGR1 intervention significantly improved the expression of vascular remodeling-related proteins. NGR1 reversed the expression of glycolysis-related genes. NGR1 reduced the levels of glycolysis-related metabolites. Further analysis revealed that NGR1 treatment decreased PFKL, HK2, and LDHA protein expression and lowered lactate levels in lung tissue. Our findings demonstrate that NGR1 effectively alleviates the pathological features of HPH in rats. NGR1 inhibits hypoxia-induced glycolysis-mediated pulmonary artery remodeling, mitigates vascular endothelial damage, and suppresses the abnormal proliferation of smooth muscle cells and fibroblasts.

## 1. Introduction

Pulmonary hypertension (PH) is a progressive obstructive pulmonary vasculopathy with diverse causes and an alarmingly high mortality rate [1]. Hypoxic pulmonary hypertension (HPH) categorized as the third subtype in the latest clinical classification of PH arises primarily from chronic obstructive pulmonary disease, interstitial lung disease, sleep-disordered breathing, alveolar hypoventilation, prolonged exposure to high altitudes, developmental abnormalities, and other contributing factors [2]. Despite its clinical significance, there is currently no effective treatment for HPH. Although long-term oxygen therapy remains the preferred approach, its extended duration and limited efficacy result in poor patient adherence [3, 4]. Furthermore, targeted therapies for other forms of PH, such as phosphodiesterase type 5 inhibitors and endothelin receptor antagonists commonly used for pulmonary arterial hypertension (Group 1 PH), have shown limited clinical benefit in HPH [5, 6]. As a result, finding effective preventive and therapeutic strategies for HPH remains an urgent and unresolved challenge.

The two hallmark pathological features of HPH—hypoxic pulmonary vasoconstriction and pulmonary artery vascular remodeling—primarily affect small pulmonary arteries [7]. Prolonged hypoxia triggers excessive proliferation of pulmonary artery endothelial cells (PAECs), causing intimal thickening, luminal narrowing, increased pulmonary vascular resistance, and elevated pulmonary artery pressure. This pressure elevation stimulates the secretion of extracellular matrix components, further thickening and remodeling the pulmonary vessels. These pathological changes create a self-perpetuating cycle that exacerbates vascular narrowing and remodeling, accelerating the progression of HPH [8]. Under hypoxic conditions, pulmonary artery smooth muscle cells (PASMCs) undergo dedifferentiation, transforming into a synthetic phenotype characterized by increased proliferation and migratory capabilities. Dedifferentiated PASMCs in distal pulmonary microarteries migrate to small arteries, where they proliferate and re-differentiate, causing irreversible thickening of the vessel wall, luminal narrowing, or even complete occlusion. These changes result in a sustained elevation of pulmonary artery pressure [9, 10]. Hypoxia also triggers rapid fibroblast proliferation and their phenotypic transformation into myofibroblasts, promoting the muscularization of previously nonmuscular arterioles and accelerating the progression of HPH [11]. Additionally, excessive extracellular matrix (ECM) deposition, along with collagen cross-linking and rearrangement, thickens the adventitia and increases vascular stiffness, further exacerbating HPH [12]. These pathological changes highlight the importance of targeting pulmonary artery vascular remodeling as a strategy to develop effective treatments for HPH.

Traditional Chinese medicine (TCM) extracts have shown promising therapeutic potential in treating HPH. Extracts such as procyanidin B2 [13], berberine [14], and resveratrol [15] from TCM have shown significant potential in preventing and treating HPH. These extracts have been found to exert anti-inflammatory effects by regulating glycolysis [16–18]. Similarly, Notoginsenoside R1 (NGR1), a key bioactive compound in Sanqi, plays an essential role in mitigating cardiopulmonary hypoxia [19]. While previous studies have indicated that NGR1 can alleviate high-altitude pulmonary edema [20, 21], its effects on HPH remain unexplored. In this study, we aimed to investigate the therapeutic potential of NGR1 in treating HPH and to elucidate its underlying molecular mechanisms using transcriptomic and untargeted metabolomic analyses. We first established a rat model of HPH under hypoxic conditions and administered NGR1 to evaluate its effects on HPH pathology. Thereafter, we conducted transcriptomic and metabolomic studies on lung tissues from the experimental groups to identify key molecular targets and pathways influenced by NGR1. We further validated these results to offer a preliminary understanding of the molecular mechanisms through which NGR1 exerts its therapeutic effects. This study is the first to demonstrate the efficacy of NGR1 in treating HPH and to explore its preliminary molecular mechanisms. Our findings offer a valuable reference for future in-depth studies on the mechanisms of NGR1 in HPH and present NGR1 as a promising candidate for clinical applications in HPH treatment.

## 2. Materials and Methods

### 2.1. Animals and Reagents

Male SD rats (200 ± 20 g) were obtained from SPF (Beijing) Biotechnology Co., Ltd. (Experimental Animal License No. SCXK (Beijing) 2024-0001). The rats were housed in groups of five per cage in a specific pathogen-free (SPF) environment with ad libitum access to food and water. The experiment received approval from the Ethical Review Committee of Animal Experiments in Yunnan University of Chinese Medicine (Ethics Approval Number: R-062024G010). Detailed information on the reagents and consumables used in this study is provided in the Supporting Information (available here).

### 2.2. Modeling, Grouping, and Drug Administration

Sixty rats were acclimated for 1 week before being randomly assigned to six groups: a control group (CON), an HPH group, a sildenafil group (SIL), an NGR1 low-dose group (L-NGR1), an NGR1 medium-dose group (M-NGR1), and an NGR1 high-dose group (H-NGR1). The control group was maintained under normal conditions, while the other groups were exposed to a hypoxic environment for 8 h per day to induce the HPH model. Hypoxia was simulated using a plateau hypoxic chamber, with environmental parameters set to simulate an altitude of 5000 m, 10% ± 0.5% oxygen, 25°C ± 1°C, and 60% ± 10% humidity. Rats in the drug treatment groups were administered daily doses of sildenafil (30 mg/kg), NGR1 (25, 50, or 100 mg/kg), or the appropriate placebo (saline) via gavage before entering the chamber. This intervention lasted for 4 weeks. After the treatment period, we performed hemodynamic assessments and collected heart and lung tissues, as well as serum samples, for further analysis.

### 2.3. Hemodynamic Measurements and Assessment of Right Ventricular Hypertrophy

After the hypoxia period, we anesthetized the rats with sodium pentobarbital and surgically exposed the external jugular vein. The distal end of the vein was ligated using a fine cotton thread, while a loose knot was prepared at the proximal end for later use. We then created a V-shaped incision below the external jugular vein, extending toward the ventricle, and carefully inserted a catheter, advancing it gently. Hemodynamic parameters, including right ventricular systolic pressure (RVSP) and mean pulmonary artery pressure (mPAP), were recorded using the PowerLab 8/35 multichannel physiological recorder and its associated pressure transducer. Following the hemodynamic measurements, the rats were euthanized and weighed. The hearts were excised, and the RV, left ventricle (LV), and septum (S) were dissected and weighed separately. The RVHI was calculated as the ratio of RV mass to the combined mass of the LV and S [RV/(LV + S)]. We assessed the degree of right ventricular hypertrophy using both the RVHI and the right ventricle-to-body weight ratio (RV/BW).

### 2.4. Pathological Staining

Lung tissues from rats were fixed in 4% paraformaldehyde, followed by dehydration in alcohol and embedding in paraffin. Tissue blocks were sectioned into 4-μm-thick slices for hematoxylin and eosin (HE) staining. The pathological sections were examined under an optical microscope, and Image-Pro Plus software was used to calculate the percent medial wall thickness (WT%) and percent medial wall area (WA%) from the HE staining to assess pulmonary artery remodeling. The formulas used for calculation were as follows:

(1) $$\begin{array}{c} \text{WT}\%=\left[\frac{\left(\text{outer diameter}-\text{inner diameter}\right)}{\text{outer diameter}}\right]\times 100\%,\\ \text{WA}\%=\left(\frac{\text{medial wall area}}{\text{total vessel area}}\right)\times 100\%. \end{array}$$

### 2.5. Immunofluorescence

Lung tissue sections embedded in paraffin were first deparaffinized and then rehydrated prior to antigen retrieval. After quenching autofluorescence, the sections were incubated with serum for 0.5 h. Primary antibodies were then applied and incubated overnight (4°C). Next day, the sections were washed and applied with secondary antibodies before 1-h incubation (room temperature, dark). The nuclei were performed DAPI staining, and the sections were mounted. Fluorescence intensity was analyzed and photographed under a microscope. Image-Pro Plus software was used to quantify the relative fluorescence intensity of the target protein.

### 2.6. Transcriptomics Analysis

Transcriptomics analysis was conducted on rat lung tissues based on methods described in previous studies [22]. Lung tissues from the same anatomical location were collected from each rat and extracted total RNA. The RNA samples were then evaluated for purity, concentration, and integrity. Next, libraries were constructed and sequencing was performed using the Illumina platform. Differentially expressed genes (DEGs) between the HPH and CON groups, as well as between the H-NGR1 and HPH groups, were identified using DESeq2 software. The criteria for DEG selection were a fold change (FC) > 1.5 or < 0.67 and a *p* value < 0.05. Gene Ontology (GO) analysis was then performed on the DEGs.

### 2.7. Untargeted Metabolomics Analysis

Analysis was performed on rat lung tissues following protocols established in prior studies [23]. Lung tissues from the same location in each group were pulverized in liquid nitrogen, followed by the addition of methanol. After homogenization, the supernatant was diluted and analyzed via LC–MS. The raw data were processed with CD 3.3 search software and cross-referenced with the mzCloud, mzVault, and Masslist databases. Background ions were eliminated by analyzing blank samples, and normalization was performed using the total metabolite quantity from quality control (QC) samples. Identified metabolites were annotated using KEGG. PCA and PLS-DA were employed to analyze the metabolic data. Differential metabolites were identified by setting the criteria of FC > 1.5 or < 0.67, *p* value < 0.05, and VIP > 1. The differential metabolites were further subjected to KEGG analysis using MetaboAnalyst 6.0.

### 2.8. Western Blot

Lung tissue from rats was homogenized using a high-speed homogenizer and then lysed in RIPA buffer on ice for 40 min. Following lysis, protein loading buffer was added to denature the proteins. The protein concentration was detected using a BCA protein assay kit. Soon after, proteins of the same mass are separated by SDS-PAGE at a constant voltage of 90 V for 2 h. The proteins were then transferred to PVDF membranes at a constant current of 400 mA for 40 min. After transfer, the membrane was blocked with rapid blocking solution for 30 min, rinsed with TBS-T, and incubated overnight at 4°C with the corresponding primary antibody. The next day, the membrane was washed with the corresponding secondary antibody and incubated for 2 h. After further washing, the membranes were developed using ECL reagent, and protein expression was quantified using ImageJ.

### 2.9. Biochemical Indicator Detection

Serum and lung tissue samples were collected from rats in each group. The levels of NO and ET-1 were measured in both serum and lung tissue, while lactate levels were determined in lung tissue, according to the manufacturers' instructions for the respective kits. Determine the protein concentration in lung tissue, which is then used for normalization of biochemical indices.

### 2.10. Statistical Analysis

SPSS Pro was used for analysis. Data are expressed as mean ± standard deviation (SD). Normally distributed data were analyzed by homogeneous variances, *t*-test or one-way analysis of variance (ANOVA), and non-normally distributed data were analyzed by the rank-sum test. *p* values less than 0.05 were considered statistically significant.

## 3. Results

### 3.1. NGR1 Intervention Improves Pathological Symptoms in HPH Rats

**Figure 1:**
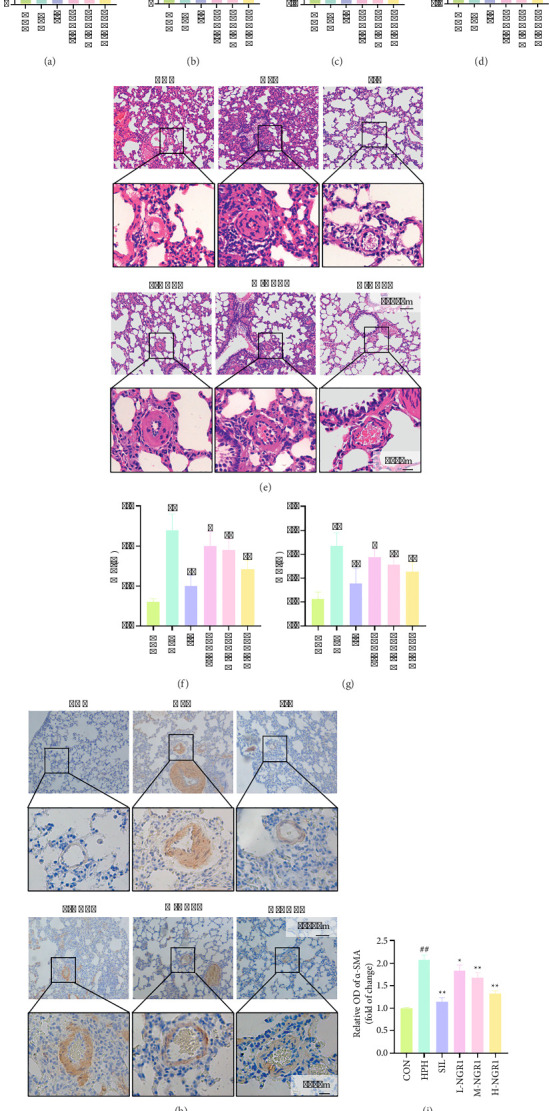
NGR1 intervention exhibits therapeutic effects on HPH rats (a, b). NGR1 intervention decreases mPAP (a) and RVSP (b) in HPH rats. (c, d) NGR1 intervention reduces RVHI (c) and RV/BW (d) in HPH rats. (e–g) HE staining reveals that NGR1 intervention improves inflammatory infiltration around the pulmonary arteries (e), and downregulates WT% (f) and WA% (g). (h, i) α-SMA immunohistochemical shows that NGR1 intervention reverses the increase in myofibroblasts in pulmonary arteries of HPH rats. Data are presented as the mean ± SD. *n* = 10. ^##^*p* < 0.01 vs. CON group; ^∗^*p* < 0.05, ^∗∗^*p* < 0.01 vs. HPH group.

Hemodynamic analysis revealed that, compared to the CON group, rats in the HPH group exhibited significantly elevated mPAP and RVSP. NGR1 treatment reduced both mPAP and RVSP in a dose-dependent manner (Figures 1(a) and 1(b)). Additionally, HPH rats showed increased RVHI and RV/BW ratios, which were normalized following NGR1 treatment (Figures 1(c) and 1(d)). Histological analysis using HE staining demonstrated that, relative to the CON group, HPH rats had greater inflammatory cell infiltration around the pulmonary arteries, as well as increased WT% and WA%. NGR1 treatment reduced inflammatory cell infiltration and decreased both WT% and WA% in the HPH rats (Figures 1(e), 1(f), and 1(g)). α-SMA immunohistochemical further revealed that, in the HPH group, lung tissues exhibited an increased proportion of collagen fibers. The α-SMA-positive area was significantly larger, and relative fluorescence intensity was markedly enhanced. NGR1 treatment reversed these pathological changes to varying extents (Figures 1(h) and 1(i)). SIL was used as a positive control, and the results showed that the high-dose NGR1 treatment had effects comparable to those of SIL. Based on these findings, we selected the H-NGR1, CON, and HPH groups for subsequent omics analysis.

### 3.2. Effects of NGR1 Intervention on the Transcriptome of HPH Rats

**Figure 2:**
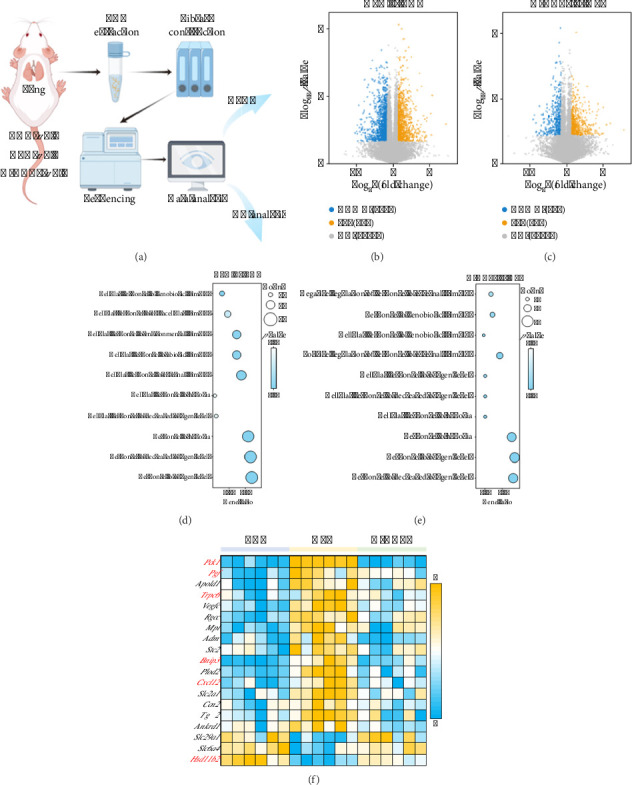
Effect of NGR1 intervention on gene expression in HPH rats. (a) Schematic diagram of transcriptomics analysis. (b, c) DEGs between HPH vs. CON (b) and H-NGR1 vs. HPH (c). (d, e) GO analysis results of DEGs between HPH vs. CON (d) and H-NGR1 vs. HPH (e). (f) Heatmap of gene expression in hypoxia-related pathways. *n* = 6.

In the transcriptomic analysis (Figure 2(a)), we applied the following criteria for screening DEGs: FC > 1.5 or < 0.67, and *p* value < 0.05. The volcano plot showed that 869 genes were upregulated and 1289 genes were downregulated in the HPH group compared to the CON group. In addition, the intervention of NGR1 resulted in the upregulation of 521 genes and downregulation of 430 genes compared to the HPH group (Figures 2(b) and 2(c)). GO analysis of these DEGs indicated that many hypoxia-related genes, such as *Pck1*, Trpc6, *Pgf*, *Bnip3*, *Cxcl12*, and *Hsd11b2*, were significantly enriched in multiple hypoxia-related pathways (Figures 2(d) and 2(e)). The expression heatmap highlighted *Pck1* as the most notably altered gene, suggesting that it may be a key gene regulated by NGR1 (Figure 2(f)).

### 3.3. NGR1 Intervention Inhibits Hypoxia-Induced Vascular Remodeling

**Figure 3:**
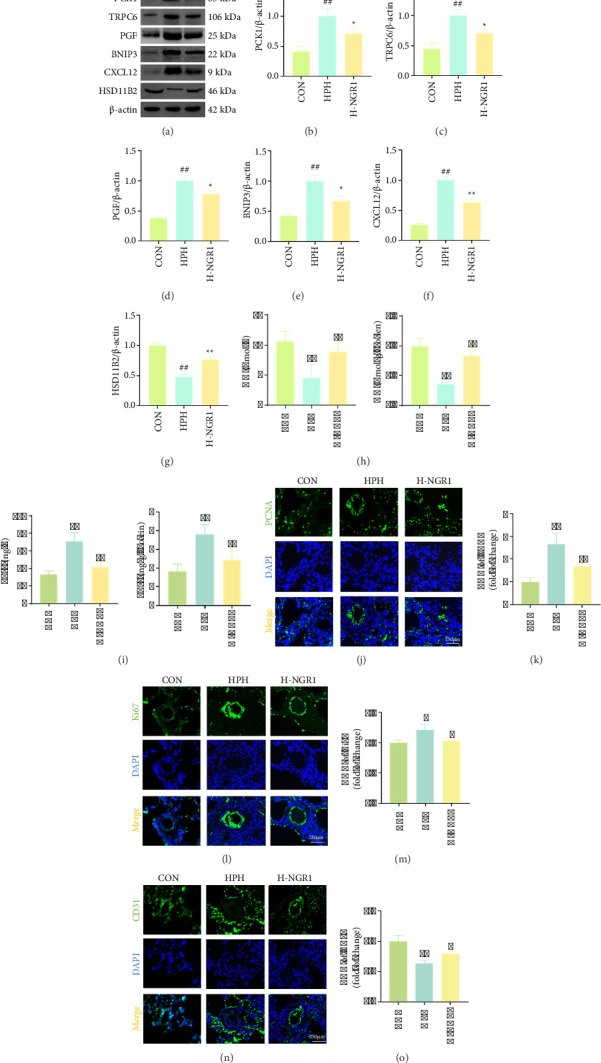
NGR1 intervention inhibits hypoxia-induced vascular remodeling. (a–g) NGR1 intervention downregulates expression of PCK1 (a, b), TRPC6 (a, c), PGF (a, d), BNIP3 (a, e), and CXCL12 (a, f) proteins, while it upregulates expression of the HSD11B2 (a, g) protein. (h, i) NGR1 intervention increases NO levels (h) and decreases ET-1 levels (i). (j–o) NGR1 intervention reduces the positive expression of PCNA (j, k) and Ki67 (l, m), and increases the positive expression of CD31 (n, o). *n* = 3.

It has been established that genes such as *Pck1*, *Trpc6*, *Pgf*, *Bnip3*, *Cxcl12*, and *Hsd11b2* are involved in hypoxia-induced vascular remodeling [24–29]. We then conducted the Western blot experiment. The results indicated that, compared to the CON group, the expression of PCK1, TRPC6, PGF, BNIP3, and CXCL12 proteins was significantly elevated in the HPH group, while HSD11B2 protein expression was significantly reduced. NGR1 treatment significantly restored the expression of these proteins (Figures 3(a), 3(b), 3(c), 3(d), 3(e), 3(f), and 3(g)). Additionally, we observed a significant decrease in NO levels and an increase in ET-1 levels in the serum and lung tissues of HPH rats (Figures 3(h) and 3(i)). The expression of PCNA and Ki67, markers of cell proliferation, was significantly elevated, while the expression of CD31, a marker of endothelial cells, was significantly reduced. However, NGR1 treatment markedly improved these parameters, reversing the pathological changes (Figures 3(j), 3(k), 3(l), 3(m), 3(n), and 3(o)).

### 3.4. Effects of NGR1 Intervention on Metabolites in HPH Rats

**Figure 4:**
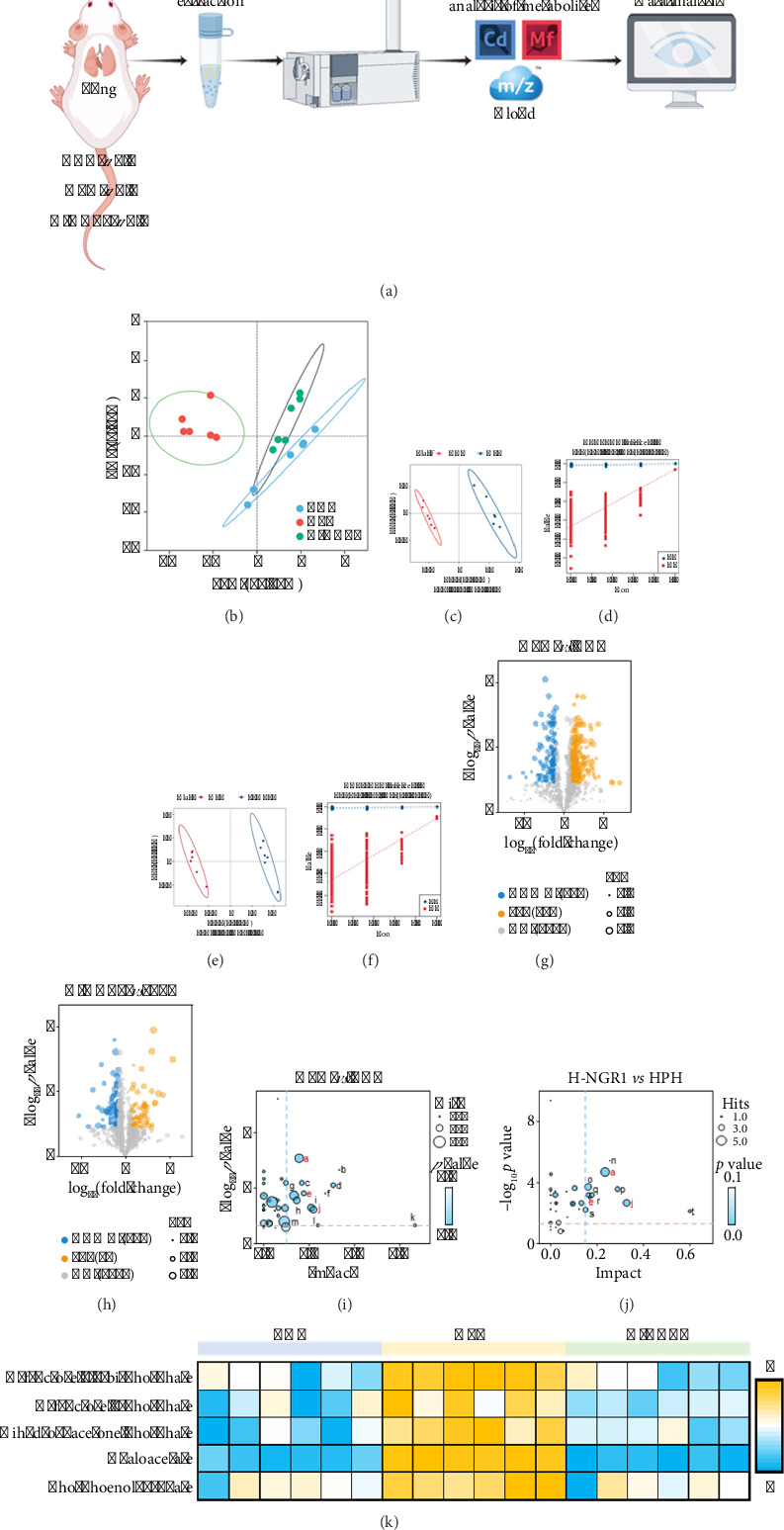
Effect of NGR1 intervention on metabolites in HPH rats. (a) Schematic diagram of untargeted metabolomics analysis. (b) PCA analysis showing significant differences in the metabolic profiles among the CON, HPH, and H-NGR1 groups. (c–f) PLS-DA analysis results indicating good fitting and predictive ability of statistical models for HPH vs. CON (c, d) and H-NGR1 vs. HPH (e, f). (g, h) Differential metabolites between HPH vs. CON (g) and H-NGR1 vs. HPH (h). (i, j) KEGG pathway enrichment analysis results of differential metabolites for HPH vs. CON (i) and H-NGR1 vs. HPH (j). (k) Heatmap of the expression of metabolites related to glycolysis/gluconeogenesis pathway. *n* = 6. KEGG pathways with significant differences are represented by (A–T), A: glycolysis/gluconeogenesis; B: riboflavin metabolism; C: glycine, serine, and threonine metabolism; D: histidine metabolism; E: pentose and glucuronate interconversions; F: ascorbate and aldarate metabolism; G: cysteine and methionine metabolism; H: pentose phosphate pathway; I: glutathione metabolism; J: fructose and mannose metabolism; K: phenylalanine, tyrosine, and tryptophan biosynthesis; L: phenylalanine metabolism; M: tyrosine metabolism; N: vitamin B6 metabolism; O: tryptophan metabolism; P: alanine, aspartate and glutamate metabolism; Q: arginine and proline metabolism; R: arginine biosynthesis; S: citrate cycle (TCA cycle); T: taurine and hypotaurine metabolism. Shared pathways between HPH vs. CON and H-NGR1 vs. HPH are marked in red.

Transcriptomic analysis highlighted the crucial role of *Pck1*, a key enzyme in glycolysis, which is involved in both glycolysis and hypoxia-induced vascular remodeling [30, 31]. To further investigate this, we performed untargeted metabolomics analysis on lung tissues from the CON, HPH, and H-NGR1 groups (Figure 4(a)). PCA and PLS-DA revealed significant metabolic differences among the groups, with clear separation observed between the CON, HPH, and H-NGR1 groups. The statistical models demonstrated robust fitting and predictive capabilities (Figures 4(a), 4(b), 4(c), 4(d), 4(e), and 4(f)). For the untargeted metabolomics analysis, we applied the following criteria to identify differential metabolites: *p* value < 0.05, VIP > 1, and FC > 1.5 or < 0.67 (Figures 4(g) and 4(h)). KEGG pathway enrichment analysis of these metabolites revealed that glycolysis/gluconeogenesis, Pentose and glucuronate interconversions, and fructose and mannose metabolism were shared pathways between the HPH vs CON and H-NGR1 vs HPH comparisons (Figures 4(i) and 4(j)). Given the significant alteration in *Pck1* expression observed in the transcriptome, and its direct involvement in glycolysis, we focused on the glycolysis/gluconeogenesis pathway. Expression analysis of related metabolites showed that NGR1 intervention downregulated the expression of D-fructose 6-phosphate, D-fructose 1,6-bisphosphate, dihydroxyacetone phosphate, phosphoenolpyruvate, and oxaloacetate (Figure 4(k)).

### 3.5. NGR1 Intervention Inhibits Glycolysis in HPH Rats

**Figure 5:**
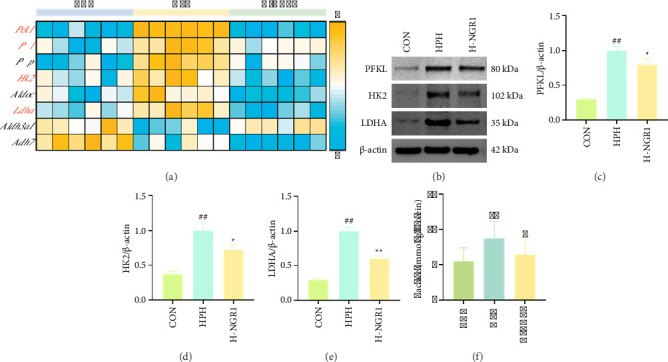
NGR1 intervention inhibits glycolysis under hypoxic conditions. (a) Heatmap of gene expression related to glycolysis/gluconeogenesis pathway. (b–e) NGR1 intervention downregulates the expression of PFKL (b, c), HK2 (b, d), and LDHA (b, e) proteins. (f) NGR1 intervention decreases the level of lactate in lung tissue. *n* = 3 for (b–f).

Transcriptome analysis revealed that genes associated with glycolysis and gluconeogenesis showed distinct changes in expression, with NGR1 downregulating *Pck1*, *Pfkl*, *Hk2*, and *Ldha* (Figure 5(a)). To validate these findings, we performed Western blot analysis to assess the protein levels of key factors. To validate these findings, we assessed the protein expression levels of these key genes. The results demonstrated a significant increase in the expression of PFKL, HK2, and LDHA proteins in the lung tissue of HPH rats compared to the CON group. However, NGR1 intervention significantly reduced the expression of these proteins (Figures 5(b), 5(c), 5(d), and 5(e)). Additionally, NGR1 treatment also decreased the lactate levels in the lung tissue (Figure 5(f)).

## 4. Discussion

HPH is an incurable, progressive vascular disease affecting the pulmonary artery. Hypoxia-induced pulmonary vascular remodeling contributes to pulmonary artery obstruction, inflammation, and fibrosis, which significantly worsen the progression of HPH [32]. In this research, we used a high-altitude hypoxic chamber to simulate a hypobaric hypoxic environment and induce HPH. Rats exposed to hypoxia developed symptoms such as increased pulmonary artery pressure, inflammatory infiltration around the pulmonary artery, enhanced collagen deposition, compensatory right ventricular hypertrophy, and decreased right ventricular contractile function [33]. These symptoms closely resemble the pathological features of HPH, confirming successful model establishment. Following NGR1 intervention, these pathological symptoms in HPH rats improved to varying degrees, suggesting that NGR1 has therapeutic potential for HPH. Furthermore, SIL is commonly used to treat PH and is often used as a positive control in HPH studies [34]. Our findings indicated that high-dose NGR1 treatment yielded results comparable to sildenafil, further supporting the potential of NGR1 as a promising therapeutic candidate for HPH.

In our exploration of the molecular mechanisms underlying NGR1 treatment for HPH through transcriptomic analysis, we identified a significant enrichment of hypoxia-related genes in multiple hypoxia-related pathways. Among these genes, PGF is an angiogenic growth factor [35]. Under normal conditions, PGF is expressed at low levels in lung tissue [36]. However, hypoxia induces a marked increase in PGF expression [37], which promotes angiogenesis in a dose-dependent manner [26]. Previous studies have shown that inhibiting PGF can mitigate the progression of HPH [27]. BNIP3, a gene induced by hypoxia, enhances cellular resistance to apoptosis and may exacerbate to vascular remodeling in HPH [27]. Research suggests that abnormal expression of CXCL12 is closely linked to impaired angiogenesis and the migration, proliferation, and differentiation of smooth muscle cells, all of which induce to vascular remodeling and exacerbate PH [28]. Neutralizing CXCL12 effectively reduces pulmonary artery hypertension in rats [38]. TRPC6, a transient receptor potential channel from the TRPC subfamily [39], is highly expressed in pulmonary vascular smooth muscle cells (PVSMCs) [40]. The increased expression and enhanced activity of TRPC6 are crucial in the progression of PH and cardiac hypertrophy [41, 42]. Inhibiting TRPC6 can significantly reduce the abnormal proliferation of PASMCs in HPH [43]. Additionally, studies have shown that the agonistic effect of ET-1 on TRPC6, combined with the inhibitory effect of NO on TRPC6, plays a crucial role in maintaining blood pressure balance [25, 44] Several studies have reported a strong association between HSD11B2 and salt sensitivity of blood pressure. A deficiency in HSD11B2 can trigger or worsen hypertension [29, 44, 45]. Notably, the substantial change in PCK1 expression in our study drew our attention. PCK1 is a key enzyme in glycolysis, closely linked to cellular metabolism, and plays a significant role in the development of diabetes and obesity [30]. Further research has shown that PCK1 promotes the proliferation and migration of PVSMCs, contributing to vascular remodeling [46]. Additionally, the hypoxia-induced upregulation of PCK1 expression correlates positively with the progression of hypertension [31]. In our study, NGR1 intervention significantly downregulated the protein levels of PGF, BNIP3, CXCL12, TRPC6, and PCK1. It also upregulated the expression of HSD11B2, increased NO levels, and reduced ET-1 levels. These findings suggest that NGR1 enhances the resistance of HPH rats to hypoxia-induced vascular remodeling. Additionally, NGR1 intervention led to the upregulation of CD31 expression in pulmonary arteries and the downregulation of PCNA and Ki67 expression, indicating that NGR1 promotes the recovery of vascular endothelial damage while inhibiting the proliferation of smooth muscle cells and fibroblasts.

Given the significant changes in PCK1 observed in the transcriptomic analysis and its essential role in glycolysis, we further investigated the molecular mechanisms of NGR1 treatment for HPH using untargeted metabolomics. Among the KEGG pathways enriched by differential metabolites, we focused on glycolysis/gluconeogenesis, which is closely associated with PCK1. Numerous studies have demonstrated that hypoxia-induced glycolysis accelerates the progression of HPH via the mediation of different factors [47]. Moreover, inhibiting the glycolysis pathway can reduce the proliferation and migration of PASMCs, thus mitigating pulmonary artery remodeling and improving HPH outcomes [48]. Key metabolites involved in glycolysis, including D-fructose 6-phosphate, D-fructose 1,6-bisphosphate, dihydroxyacetone phosphate, phosphoenolpyruvate, and oxaloacetate, are critical to this metabolic process [49]. In our study, NGR1-intervention significantly decreased these metabolite levels, suggesting that NGR1 exerts an antiglycolytic effect. Further analysis revealed that NGR1 inhibited the expression of PFKL, HK2, and LDHA, all of which are essential enzymes in the glycolytic pathway. HK2 catalyzes the conversion of glucose to D-fructose 6-phosphate, which is subsequently converted to D-fructose 1,6-bisphosphate by PFKL, facilitating the continuation of glycolysis. Studies have highlighted the potential of PFKL and HK2 as therapeutic targets for HPH [50–52]. LDHA is a key enzyme that catalyzes the production of lactate and plays a central role in vascular remodeling in HPH. Inhibiting LDHA has been proposed as a potential therapeutic strategy for improving HPH pathology [53]. Meanwhile, our results have demonstrated the inhibitory effect of NGR1 on lactate production. These findings further support the potential of NGR1 to inhibit glycolysis and its therapeutic value in treating HPH.

Our study demonstrates that NGR1 can ameliorate HPH by inhibiting glycolysis, and this effect is highly likely related to the regulation of PCK1. However, the mechanism of how NGR1 targets PCK1 remains to be elucidated. Previous studies showed that epigenetic modifications such as DNA methylation could affect the expression of PCK1 [54]. Whether NGR1 downregulated the expression of PCK1 through regulating DNA methylation can be studied in the future. Besides, CREB-regulated transcription coactivator 2 (CRTC2) has been demonstrated to regulate the transcription level of PCK1 [55]. In addition, in vitro studies can be conducted in the future to detect the direct interactions between NGR1 and glycolysis-related proteins using molecular docking, cellular thermal shift assay (CETSA), and drug affinity responsive target stability (DARTS). On the other hand, hypoxia-related genes have also been enriched according to the transcriptomics, and the effects of NGR1 on other differential genes can be validated using in vitro models to avoid the off-target effects of NGR1. Novel techniques such as single-cell sequencing and spatial transcriptomics can be used to further identify the target cell of NGR1. Importantly, studies utilizing human lung tissue samples or human-derived cells should be conducted to further confirm the therapeutic efficacy of NGR1 in humans. Moreover, pharmacokinetic analyses to investigate the metabolic profile of NGR1 across various organs, along with assessments of its safety advantages, will provide experimental evidence to support the clinical application of NGR1.

## 5. Conclusion

**Figure 6:**
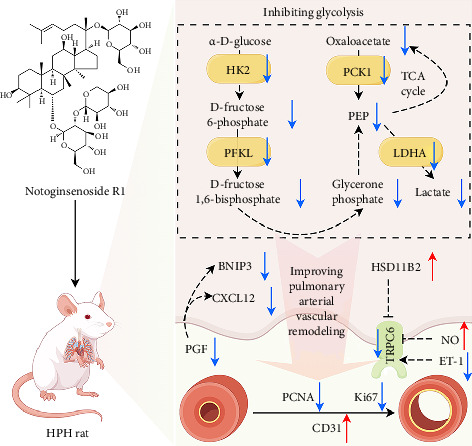
NGR1 inhibits glycolysis-mediated pulmonary artery vascular remodeling under hypoxic conditions, improves vascular endothelial damage and abnormal proliferation of smooth muscle cells or fibroblasts, and alleviates HPH (drawn by figdraw 2.0).

This study, for the first time, demonstrates the efficacy of NGR1 in treating HPH. Through a comprehensive mechanistic analysis that integrates transcriptomics and untargeted metabolomics, we show that NGR1 inhibits glycolysis-driven pulmonary artery vascular remodeling under hypoxic conditions. Additionally, NGR1 improves vascular endothelial damage and reduces the abnormal proliferation of smooth muscle cells and fibroblasts, thereby alleviating HPH (Figure 6). These findings provide valuable insights into the molecular mechanisms by which NGR1 acts in HPH treatment and highlight its potential as a promising therapeutic candidate for clinical application in HPH management.

## Data Availability

The data that support the findings of this study are available from the corresponding author upon reasonable request.

## Supporting Information

Reagents: Detailed information on the reagents and consumables used in this study.
