# Supplementary material for: Notoginsenoside R1 Improved Hypoxic Pulmonary Hypertension by Inhibiting Glycolysis-Mediated Pulmonary Arterial Vascular Remodeling
Source: Can Respir J. 2025 Oct 13;2025:2884885. doi: 10.1155/carj/2884885 (PMC12537236; doi:10.1155/carj/2884885)
Supplement: Supporting Information — Additional supporting information can be found online in the Supporting Information section. [file 2884885.f1.docx]

***Supplementary Material***

**Reagents**

Sildenafil (UK-92480) were purchased from Shanghai Medchemexpress Bio-Technology Co., Ltd. (Shanghai, China). HE staining assay kit ([D006-1-1](http://www.njjcbio.com/products.asp?id=476)) were purchased from Nanjing Jiancheng Biological Engineering Institute (Nanjing, China). NO(A013-2-1), ET-1(H093-1-2), BCA(A045-4-2) were purchased from Nanjing Jiancheng Biological Engineering Institute (Nanjing, China). PCK1(12940s), BNIP3(3769s), CXCL12(3740s), PFKL (89495s) were purchased from Cell Signaling Technology (Shanghai, China). HSD11B2(ab203132) were purchased from Abcam (Shanghai, China). TRPC6(bs-21380R) were purchased from bioss Bio-Technology Co., Ltd. (Beijing, China). PGF(PA5-79814), HK2(MA5-14849), LDHA(PA5-27406) were purchased from Thermo Fisher Scientific Bio-Technology Co., Ltd. (Shanghai, China). PCNA(ab29), Ki67(ab15580)，CD31(ab9498) were purchased from Abcam (Shanghai, China).
